# Exploring the factors influencing continuance intention to use simulation software in mechatronic engineering by integrating the TAM and TTF model

**DOI:** 10.1038/s41598-025-90585-0

**Published:** 2025-04-08

**Authors:** Jiaxing Li, Wenhong Liang

**Affiliations:** 1 Philosophy Department, School of Humanities and Social Science, Xi’an Jiaotong University, Xi’an, 710049 China; 2 Present Address: School of Mechatronic Engineering, Xi’an Technological University, Xi’an, 7100221 China

**Keywords:** Psychology, Engineering, Information technology

## Abstract

This study aims to investigate the factors influencing students’ continuance intention to use simulation software in mechatronic engineering education by integrating the technology acceptance model (TAM) and task technology fit (TTF) model. This study distributed questionnaires and collected data through the WJX platform. The questionnaire incorporated established and validated scales to measure key constructs, including perceived ease of use, perceived usefulness, task‒technology fit, student satisfaction, and continuance intention. The participants were 351 mechatronics engineering students (including undergraduates, postgraduates, and doctoral students) from universities in Shaanxi Province, China, who had some experience using simulation software. The data were analysed via structural equation modelling (SEM) to ensure that the sample size matched the number of model variables. The results indicated that perceived ease of use, perceived usefulness, and task‒technology fit positively affected students’ satisfaction with the simulation software. Task‒technology fit and satisfaction have a positive effect on the students’ continuance intention to use the simulation software. Notably, the effects of task‒technology fit and satisfaction on students’ willingness to persist in using the simulation software are approximately similar. In addition, task‒technology fit not only directly influenced the willingness to persist in using simulation software in mechatronics learning but also indirectly affected these intentions through students’ satisfaction with the simulation software. These findings provide valuable insights into the design and implementation of simulation software in mechatronics engineering education, emphasizing the importance of aligning software functions with students’ learning tasks and goals.

**Supplementary Information:**

The online version contains supplementary material available at 10.1038/s41598-025-90585-0.

## Introduction

In the area of mechatronic engineering education, practical teaching plays an important role in cultivating the innovative ability of students, and the smooth implementation of practical teaching helps improve the quality of talent cultivation. However, traditional experimental teaching and practical teaching cannot meet the requirements of experimental practice teaching in this new situation^1^. On this basis, a new teaching and study tool—simulation software—is being used in mechanical engineering education, playing an increasingly important role in teaching and study. Simulation software can help students understand theoretical knowledge^2^, engage in virtual experiments and design projects^3^, enhance practical application skills^3^, and improve problem-solving abilities^2^. Students can simulate the operation of various mechanical, electrical, and control systems in simulation software to experience and understand the operation and effects of actual engineering projects^4^. Teachers can use simulation software to design virtual experiments and project tasks, enabling students to engage in practical operations in a simulated environment. Students can use different design schemes and parameter settings, analyse the results, optimize designs, and learn practical skills in a safe environment.

As computer simulations have become more popular in mechatronic engineering education, some scholars have begun researching the role of simulation software in practical teaching in mechanical and electronic engineering programs. For example, Hamrol highlighted in “Lecture Notes in Mechanical Engineering” various practical applications of simulation technology in mechatronics engineering education^5^. Shuvra Das used his book “Modeling and Simulation of Mechatronic Systems using Simscape” to guide students in learning how to develop mechatronic system models with Simscape (a MATLAB tool box). He also used many examples from different engineering domains to demonstrate how to develop mechatronic system models and what type of information can be obtained from the analyses^6^. In summary, simulation software has become an indispensable tool in mechatronic engineering education. However, to the best of our knowledge, no prior study has investigated the continuance intention of students towards using simulation software for their mechatronic engineering study, which holds significant value for the design and continued promotion of simulation software and the development of teaching strategies in mechatronic engineering education, making the insights from this study valuable at the theoretical and practical levels.

## Literature review

### Simulation software

Simulation software simulates the operation and behaviour of real systems via mathematical models and computational algorithms, providing visual results and data analysis^7^. In recent years, simulation software has been used in an increasingly wide range of fields, such as aerospace, automotive, and electronics, to simulate the operation and performance of complex systems. In the field of engineering, simulation software is widely used, including but not limited to structural analysis, fluid dynamics simulation, heat conduction analysis, and electromagnetic field simulation, to simulate the performance and behaviour of systems. Using simulation software for structural analysis, such as simulations for earthquake engineering^8^ and simulations of optimized concrete girders^9^, helps engineers evaluate the strength and stability of structures. Fluid dynamics simulations can simulate the flow characteristics of fluids such as air and water, providing crucial insights for designing aircraft, cars, and pipelines. Electromagnetic field simulations are used to simulate the behaviours of electromagnetic fields in various devices and systems, such as motors and transformers, contributing to optimizing electromagnetic designs^3^, such as evaluating the performance of wearable antennas^10^. Through simulation software, engineers can conduct virtual experiments, test design schemes, optimize product performance, reduce costs and risks, and improve the efficiency and accuracy of engineering design.

In mechatronic engineering education, commonly used simulation software in teaching includes Alitium, Protues, MATLAB, CFX, Abques, and Multisim. Existing research mainly discusses modelling methods based on various simulation software. For example, Mohd and Makoto proposed a modelling and simulation approach using stereotypes and specializations of SYSML standards to facilitate mechatronics system design. aiming to bridge the gap between systems design and system simulation in the context of mechatronics^11^. Another area of existing research has analysed the use of simulation software in mechanical engineering education from the perspective of technological characteristics. For instance, Kenjo explained why simulation software serves as a critical reference in engineering education from the vantage point of undergraduate education and the retraining of technical instructors and working engineers^12^. Additionally, Ashkan presented an overview of current applications and the ongoing transition from physical experimentation to digital simulations and immersive simulated learning environments in engineering education. He also discussed how the immersive simulation-based learning (ISBL) approach provides a framework to reuse models developed during simulation projects for educational purposes^13^.

### Technology acceptance model (TAM)

Although simulation software has become indispensable in engineering education for its ability to simulate complex systems and provide valuable insights through virtual experiments and performance evaluations, its continued use often depends on students’ and educators’ assessments of its usefulness and ease of use—two key factors that can be elucidated through the technology acceptance model (TAM). The technology acceptance model (TAM) is a theoretical framework that helps in understanding how users adopt and use new information technology. Developed by Fred Davis in the 1980s, the TAM focuses on the behavioural intention to use a technology on the basis of perceived usefulness and perceived ease of use^14^. Several key components of the TAM are as follows:

1. Perceived usefulness (PU): This refers to the extent to which a user believes that using a particular technology will enhance their performance or productivity. Users are more likely to accept a technology if they perceive it as useful in their work or daily life^14^.
2. Perceived ease of use (PEU): This refers to the degree to which a user believes that using a technology will be free of effort. If a technology is perceived as easy to use, users are more likely to adopt it^14^.
3. Behavioural intention to use (BI): This refers to users’ intention to use a technology based on their perceptions of usefulness and ease of use. The stronger the intention to use is, the more likely the user is to actually use the technology^15^.
4. Actual use (AU): This refers to the measure of the actual use of the technology by the user. It is influenced by the user’s intention and external factors that may affect the adoption of the technology^15^.

TAM can be applied in various contexts, including educational technology^16,17^, e-commerce^18^, and healthcare systems^19^, to understand user acceptance and adoption of new technologies. It is often used by researchers and practitioners to design and evaluate technology initiatives to ensure successful adoption and implementation. Overall, the TAM provides a valuable framework for studying the factors that influence user acceptance of technology, which aims to delve into user attitudes and decision-making processes concerning novel technological advancements and guide R&D personnel in developing user-friendly and effective technological solutions^20^. Therefore, many researchers have combined the TAM with other models or theories; for example, Ming Yang combined the TAM with the information systems success model (IS success model) to develop a theoretical model for studying learners’ continuance intentions towards participation in MOOCs^21^. In addition, scholars have discussed the theory of reasoned behaviour, technology acceptance model, motivation model, theory of planned behaviour, unified model of technology acceptance and planned behaviour, PC use model, diffusion theory, and social cognitive theory. Their deficiencies and strengths were compared, leading to the development of the unified theory of acceptance and use of technology (UTAUT)^22^.

### Task‒technology fit (TTF) model

The task‒technology fit (TTF) model is a theoretical framework that focuses on assessing the match between the characteristics of a task and the characteristics of a technology. Developed by Goodhue and Thompson in the 1990s^23^, the TTF model is used to evaluate how well a technology supports the requirements and objectives of a specific task or set of tasks. There are several key components of the task‒technology fit model:

1. Task characteristics (TAC): These include the nature of the task, its complexity, the information processing needed, and the goals and objectives of the task. Understanding task characteristics is essential for assessing the technology requirements needed to support a task effectively^23^.
2. Technology characteristics (TEC): These refer to the features and capabilities of the technology being used or considered for the task, including factors such as usability, functionality, compatibility, and scalability of the technology^23^.

According to TTF theory, a fit assessment is used to evaluate the fit between task characteristics and technology characteristics. A good fit indicates that the technology aligns well with the task requirements and can support the tasks efficiently. The TTF model assesses how well the technology enables users to perform tasks effectively and efficiently. It also considers user satisfaction with the technology in supporting task performance^24^. User attitude is affected by the fit between individuals and technology, whereas technology performance is affected by the fit between tasks and technology and between tasks and individuals. Users of technology who perceive it as fitting their individual needs can view it as more useful than it actually is in terms of improving task performance. Finally, technology performance translates into task performance^24^. In other words, by assessing the fit between tasks and technologies, organizations can make informed decisions about technology adoption, implementation, and optimization to enhance overall performance and productivity. In addition, unlike other theories and models that focus on users’ intentions to continue using technology, TTF theory emphasizes the practical aspects of technology use in daily life and the alignment between technology and task characteristics. Consequently, even though a technology may be perceived as being advanced, if it does not fit users’ task requirements, they may not adopt it^25^.

With respect to the fields in which the TTF model is commonly used, studies have shown that the TTF model is widely used in the areas of information systems and technology management to evaluate the effectiveness of technology solutions in meeting organizational and user needs^26^. For example, Ranvir S. Rai conceptualized task‒technology fit as how well a technology is integrated with a set of interrelated tasks to study the actual use of digital textbook services, a type of digital learning technology^27^. Arjan Raven’s study on the TTF model using digital videos for presentations revealed a positive impact on skill and task fit, which can enhance student performance in various settings^28^.

### The integrated model of the TAM and TTF

Although the TTF model and the TAM have garnered considerable academic interest and have been supported for their ability to explain and predict outcomes effectively, several shortcomings have been identified. The TAM focuses primarily on perceived usefulness (PU) and perceived ease of use (PEU) as the main determinants of user acceptance, but it does not consider other potential factors, such as the characteristics of technology, the complexity of technology, and personal traits that might also influence technology acceptance^29^. Even though the TTF model emphasizes the importance of fit between tasks and technology, it does not provide a coherent understanding of what constitutes a task environment and how such an environment affects adoption in a multifaceted context in which the interrelatedness between tasks might be high^27^.

The integrated model of the TAM and TTF is a sophisticated conceptual framework that merges two established theories in the information systems field to enhance the comprehension of user acceptance and adoption of technology. This model offers a comprehensive outlook on technology acceptance by not only considering the perceived ease of use and usefulness (from the TAM) but also evaluating how effectively the technology aligns with the tasks it is designed to facilitate (from the TTF model). By incorporating the alignment between the TAM and the TTF model, the combined model can more precisely forecast user acceptance and willingness to use a technology. It serves as a valuable guide for both research and practical applications in technology acceptance, encompassing both individual perceptions and task-related components. The model integrates various factors that influence technology acceptance, presenting a more nuanced perspective than models that focus solely on perceived usefulness and ease of use or the attributes of technology and individuals^30^.

Previous studies have shown that the research framework for integrating the TAM for adoption and the TTF model for utility provides a more comprehensive understanding of the behaviours related to this context^21^. Currently, many scholars have employed a combined model of TAM and TTF to investigate the adoption of a new technology, users’ intention to continue using a technology, and factors influencing users’ acceptance and utilization of a particular technology. For example, Bing Wu proposed a unified model integrating the TAM, TTF model, MOOC features and social motivation to investigate continuance intention to use MOOCs^21^. Ranvir S. Rai tested the integrated model of the TAM and the TTF model in a field study and investigated the actual use of digital textbook services as a type of digital learning technology^27^. Chengliang Wang examined TAM and TTF theory and conducted an empirical analysis of user satisfaction with new online learning spaces^21^. The findings from these prior studies serve as a source of inspiration for the current study, influencing the direction and focus of our investigation. The combination of the TAM and TTF model can not only explain why simulation software is widely accepted by students in the field of mechatronics engineering education, focusing on variables related to the technical or functional aspects of the system that affect individuals’ intentions to use it, but also examine the connection and relevance between simulation software and learning tasks, thereby enabling the assessment of when and why students use simulation software for learning activities. Therefore, this study aims to investigate the continuance intention to use simulation software in mechatronic engineering education on the basis of the aforementioned theoretical models. This study attempts to explore the impact and role of student characteristics, simulation software features, perceived usefulness, and ease of use of simulation software on students’ satisfaction with and continuance intention to use simulation software in mechatronics engineering education.

## Research design and hypotheses

### Research model

**Fig. 1:**
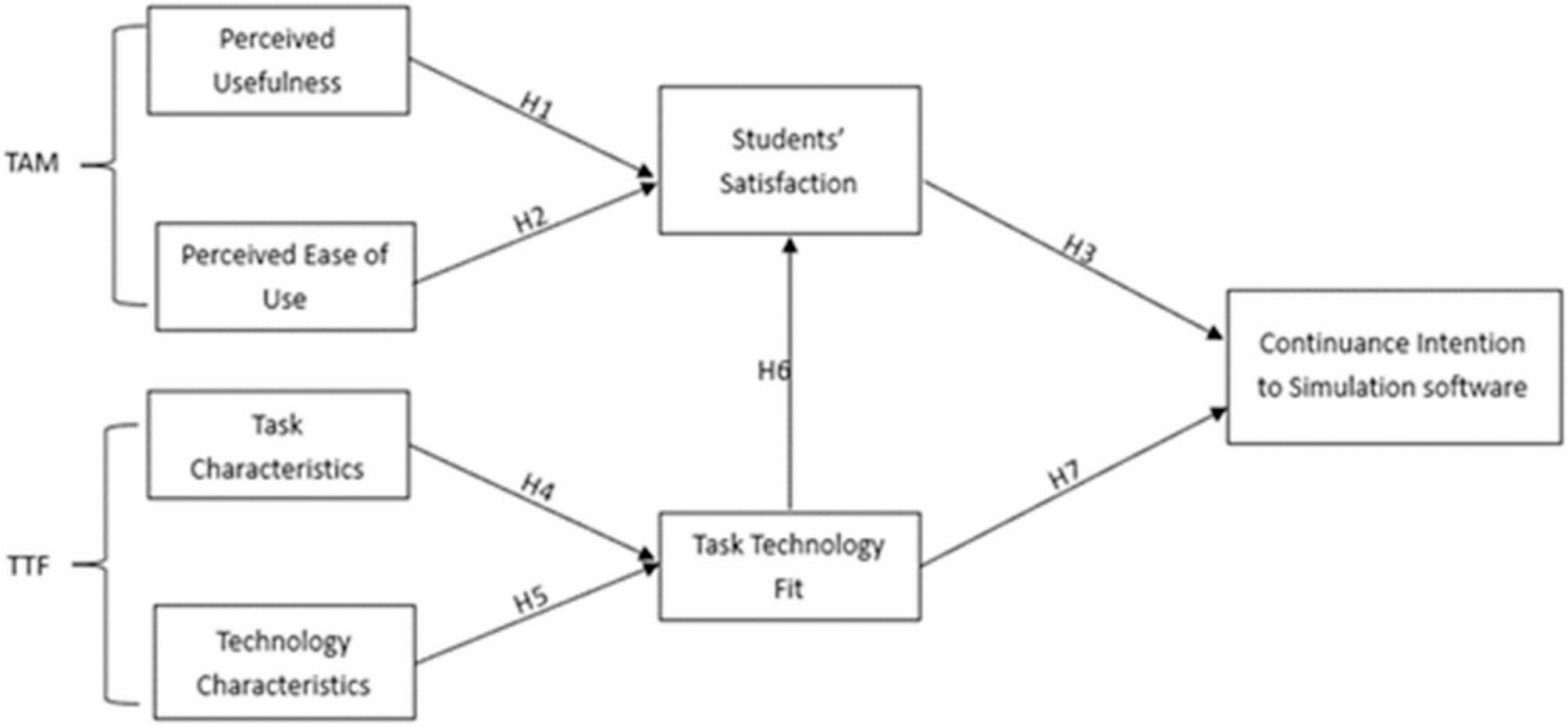
Proposed research model.

In our study, while exploring the continuance intention of students’ use of simulation software in mechatronic engineering, we found that focusing solely on the acceptance of students towards this technology was insufficient. We also need to consider the alignment between the characteristics of students’ needs in mechatronics engineering education and the functionalities of simulation software as a learning tool. The TTF model, which considers the impact of the alignment between task characteristics and technology characteristics on user behaviour^23^, fulfils the need to study the continuance intentions of students’ use of simulation software in mechatronic engineering. Therefore, we inferred that combining the TAM and TTF model would provide a better framework for understanding these aspects. On the basis of these two models, we identified several latent variables as influential factors in predicting the continuance intentions of students’ use of simulation software in mechatronic engineering. On the basis of existing research, a research model was proposed for this study, as shown in Fig. 1.

#### Perceived usefulness

Perceived usefulness is a measure of the degree to which a person believes that using a particular system would enhance their job performance. This suggests that the more useful a technology is perceived to be, the more likely it is to be accepted^14^. This variable can be defined as the degree to which students’ learning or research outcomes improve when simulation software is used as a learning or research tool. Previous studies have indicated that the perception of usefulness serves as a potent and direct determinant of satisfaction with the learning experience^31^. Thus, if students can achieve better results in their study and research in the field of mechatronics engineering through the use of simulation software, they will be more satisfied when using the software and show more intention to continue. Therefore, the following hypothesis was formulated:

##### Hypothesis 1

(H1): In mechatronic engineering education, perceived usefulness has a positive effect on student’ satisfaction with simulation software.

#### Perceived ease of use

Perceived ease of use refers to the degree to which a person believes that using a particular system would be free of effort^14^ or the amount of effort an individual spends in using a technology^32^. The easier a technology is to use, the more likely it is to be accepted^14^. Prior studies have demonstrated that perceived ease of use has a direct effect on learners’ satisfaction with new technologies^33^. Many studies have demonstrated that perceived ease of use can directly impact the satisfaction of users with a technology^34^. Therefore, the following hypothesis was formulated:

##### Hypothesis 2

(H2): In mechatronic engineering education, perceived ease of use has a positive effect on student’ satisfaction with simulation software.

#### Students’ satisfaction

On the basis of prior research findings, user satisfaction is widely understood to be an attitude held by individual users^35^; therefore, we conceptualize students’ attitudes towards simulation software as stemming from the satisfaction they experience during its use. Satisfaction, a psychological construct linked to subjective emotions, is commonly defined in the educational context as the feeling of contentment and accomplishment within a specific learning setting^36^ and is positively correlated with intention^37^. The notion of attitude is a theoretical latent variable, and evaluating attitudes can potentially introduce cognitive bias. Attitude towards use is determined by perceived usefulness and perceived ease of use, and a positive attitude towards using a technology is a significant predictor of the intention to use that technology. Therefore, students’ satisfaction is a crucial factor in predicting the intentions of students to continue their education^38^. Therefore, we propose the following hypothesis:

##### Hypothesis 3

(H3): In mechatronic engineering education, students’ satisfaction has a positive effect on the continuance intention to use simulation software.

#### Task characteristics

Task characteristics refer to the attributes related to tasks performed via a specific technology. These features can significantly influence users’ acceptance and willingness to use the technology^23^. If the technology is closely related to the user’s work goals and tasks, the user is more likely to accept and use the technology^39^. Therefore, the following hypothesis is formulated:

##### Hypothesis 4

(H4): In mechatronic engineering education, task characteristics positively influence the task‒technology fit of simulation software.

#### Technology characteristics

Technology characteristics refer to the inherent qualities and features of a technological system or tool that can influence its acceptance, use, and success in various contexts^23^, including the functionality and usefulness of the technology in achieving specific goals or tasks and the degree to which the technology fits with the user’s existing systems, processes, and preferences^40^, which helps ensure that the technology meets the needs of the users and increases the likelihood of successful adoption and use. In accordance, we suggest the following hypothesis:

##### Hypothesis 5

(H5): In mechatronic engineering education, technology characteristics positively influence the task‒technology fit of simulation software.

#### Task‒technology fit

When discussing the acceptance and satisfaction of a new technology in industries or professions that have specific requirements for technology, the alignment of tasks with the technology is a crucial indicator^41^. For example, Hsiao’s study explored the task‒technology fit of mobile nursing information systems in relation to nursing performance^41^. Education in the field of mechatronic engineering has specific demands for professionalism and the relevance of learning tools; hence, studies on the satisfaction and continuance intention of students towards using simulation software need to consider the alignment of technology with tasks. Task‒technology fit emphasizes the alignment between the specific characteristics of a technology and the requirements of the task^23^, which affects the satisfaction and continuance intentions of users^42^. Therefore, we suggest the following hypotheses:

##### Hypothesis 6

(H6): In mechatronic engineering education, task‒technology fit has a positive effect on students’ satisfaction with simulation software.

##### Hypothesis 7

(H7): In mechatronic engineering education, task‒technology fit has a positive effect on the continuance intention of students towards the use of simulation software.

### Research instrument

Following the foundational principles of structural equation modelling (SEM) research design, we constructed questionnaires for our study participants by incorporating established and validated scales^43^. Owing to the limited research on students’ continuance intention to use simulation software in mechatronic engineering education and the absence of complex questionnaires in this context, we refer to the questionnaire designs developed by prominent scholars in the field of continuance intention towards new e-learning spaces^44^.

Several factors, including the characteristics of the simulation software and the students’ conditions and requirements during their mechatronic engineering education, were considered when crafting the questionnaires and choosing the questionnaire items. Drawing from this foundation, we utilized the Delphi method^45^ and crafted an initial questionnaire draft following extensive dialogues with experts in the fields of education and mechatronic engineering. In this research, we utilized a consistent seven-point Likert scale. The original questionnaire included 27 questions covering seven underlying factors. Before the main study, a pilot test was conducted on the questionnaire. In terms of the ration of observations to variables, the general rule is to have a minimum of five times as many observations as the number of variables to be analyzed, and a more acceptable sample size would have a 10:1 ratio^46^. That is to say, each observed variable requires at least 5 to 10 samples. Therefore, the sample size needed for the pilot test is between 35 and 70. During this phase, 61 responses were gathered and analysed. Following the analysis, 3 questions with low reliability and factor loadings were removed from the questionnaire. Furthermore, some new questions that reflect the students’ basic information related to gender, academic stage, and experience with simulation software were added to the final version of the questionnaire (Table 1).

**Table 1:** Question items and references.

Constructs	Coding	Items	References
Perceived usefulness (PU)	PU1	Using simulation software helps me to understand knowledge.	^21^
	PU2	Using simulation software helps me to practice what I learn.	
	PU3	Using simulation software can help me improve my learning (research) abilities.	^15^
	PU4	Without simulation software, it would be very difficult for me to accomplish my learning (research) tasks.	
Perceived ease of use (PEU)	PEU1	Learning how to use simulation software is easy.	^47^
	PEU2	Using simulation software is easy for me.	
	PEU3	I find it easy to recover from errors encountered while using simulation software.	^15^
	PEU4	The simulation software system provides helpful guidance in performing tasks.	
Students’ satisfaction (SS)	SS1	I am satisfied with the function of simulation software for my learning (research).	^48^
	SS2	I enjoy the learning (research) experience through using simulation software.	
	SS3	It is a wise choice to use simulation software for learning (research).	
	SS4	I believe that simulation software is an excellent learning tool.	^21^
Task characteristics (TAC)	TAC1	When I use simulation software for study, I often need to simulate various scenarios that occur during the operation of the system.	^49^
	TAC2	When I use simulation software for study, my task involves conducting simulation analysis.	
	TAC3	My study tasks require me to conduct simulation analysis on systems.	
Technology characteristics (TEC)	TEC1	Simulation software provides simulation capabilities for various operating scenarios of systems.	^49^
	TEC2	The simulation analysis function of simulation software is very useful.	
	TEC3	Simulation software can help me design or conduct simulation experiments.	
Task‒technology fit (TTF)	TTF1	The simulation software meets my needs.	^49^
	TTF2	It is easy to know which function to use during the process of using simulation software.	
	TTF3	Using simulation software is appropriate to help me with my tasks.	
Continuance intention towards simulation software (CIS)	CIS1	I would not give up using simulation software for learning (research) midway.	^44^
	CIS2	I intend to continue learning (research) through simulation software rather than using other methods.	
	CIS3	I plan to continue using simulation software in the future or at least maintain the same level of activity as I do now.	
Basic information	Gender (select one answer choice): (A) Male; (B) Female. Academic stage (select one answer choice): (A) Freshman; (B) Sophomore; (C) Junior; (D) Senior; (E) Graduate; (F) Doctoral. Simulation software commonly used by students in their research and studies (multiple choices are possible): (A) Alitium; (B) Protues; (C) MATLAB; (D) CFX; (E) Abques; (F) Multisim; (G) Others. Experience with simulation software (select one answer choice): A. Half a year; B.1 year; C. 2 years; D. 3 years; E. More than 3 years.		

### Data collection

This study is not related to medical research, does not involve clinical trials, does not include minors and is conducted anonymously. This survey does not require participants to provide any information related to privacy issues. The participants in this survey are students majoring in mechatronic engineering, including undergraduate, graduate, and doctoral students from universities in Shaanxi, China. Before they answered the questionnaire, the participants were provided with detailed information about the study. They only proceeded with the survey after providing informed consent and had the option to withdraw if desired. The survey was conducted on the WJX platform^50^, which is a professional online survey platform primarily used for creating and managing various types of questionnaires and surveys. It supports the sharing of surveys through multiple methods, such as links and QR codes, making it convenient for participants to fill them out. The platform also provides real-time data statistics and analysis features, allowing users to view results intuitively and generate reports. The survey started on June 2, 2024, and ended on June 10, 2024. To ensure the authenticity of participant information, this questionnaire is distributed in two forms regarding the distribution and response time: first, the distribution time is scheduled before the mechanical and electrical engineering courses at various universities, with prior communication with the course instructors to arrange for students to answer collectively in the classroom; second, the questionnaire is included with the students’ postclass assignments. To ensure the validity of the collected data, a preliminary question was included at the beginning of the questionnaire asking the participating students whether they regularly used simulation software (such as Alitium, Protues, MATLAB, CFX, Abques, and Multisim). Participants who did not use these software platforms were not required to proceed with the survey. The WJX platform’s answer-time recording feature was utilized to monitor and evaluate the data quality.

Following the necessary preparations, data were gathered from 360 students. To ensure the quality of the sample, three screening criteria for the questionnaires were implemented, following the methodology of Chengliang et al.^44^ These criteria include the time taken to complete the questionnaire, the correct identification of reverse-coded items, and the consistency of responses, in order to exclude invalid questionnaires. On the basis of the pilot test findings, the participants typically spent 1–3 min completing the questionnaire. After a thorough screening process, 9 inadequate questionnaires were eliminated, leaving 351 valid questionnaires for further analysis. In addition, during the preliminary testing phase, the sample size is usually between 10% and 30% of the final sample size. The effective sample number we ultimately collected proves that the sample size used in the prior pilot tests was reasonable.

In terms of sample size, Barrett recommended a sample size of at least eight times the number of model variables^51^. However, Barrett observed that the chi-square value tended to inflate when the sample size exceeded 500 with the default maximum likelihood estimation in structural equation modelling (SEM), resulting in a poor model fit. Following this guideline, a sample size of 192, which equated to eight times the number of model variables (24 in this case), was deemed necessary for this study. Therefore, the inclusion of 351 participants in this study exceeded this requirement. Among the 351 valid questionnaires, 275 were from male participants and 76 from female participants. Although there is an obvious gender imbalance, it is common in Chinese universities for there to be more male students than female students in the field of mechatronic engineering. In terms of the academic stage, the participants included 5 freshmen, 3 sophomores, 169 juniors, 135 seniors, 19 graduate students, and 20 doctoral students. Notably, students majoring in mechatronics engineering generally start using simulation software frequently after completing the foundational professional knowledge in their first and second years. Therefore, many first- and second-year students do not need to answer the following question because of their lack of experience with simulation software. Students most commonly use simulation software such as Altium, Proteus, MATLAB, and Multisim. With respect to the participants’ experience with simulation software, 12.25% of the participants had six months of experience using simulation software, 42.45% had one year of experience, 29.34% had two years of experience, 9.97% had three years of experience, and 5.98% had more than three years of experience using simulation software. Therefore, the participants are mechatronics engineering students with a certain level of experience in using simulation software.

### Ethical approval

This study is not related to medical research, does not involve clinical trials, does not include minors and is conducted anonymously. This survey does not require participants to provide any information related to privacy issues. Before they answered the questionnaire, the participants were provided with detailed information about the study. The participants in the survey had the right to withdraw at any time.

## Results

### Common method variance test

The data for this study were gathered from a single source, the participants, via a self-perception self-statement approach. This method of data collection is vulnerable to common method variance (CMV), which stems from shared measurement environments, item contexts, and characteristics, potentially creating artificial associations between predictor and criterion variables. To address this issue, we implemented the strategy recommended by Cham et al.^52^ Specifically, during data collection, distinct pages were intentionally designed for different variables in the questionnaire to provide respondents with adequate breaks between sections. This approach aims to mitigate CMV caused by repetitive use of the same scale^53^. Furthermore, Harman’s single-factor test was employed in this study, utilizing principal component analysis to investigate common method bias. Principal component analysis was applied to all the items, revealing seven factors with eigenvalues greater than one. The primary common factor explained 27.041% of the variance, which was well below the 50% threshold suggested by Hair et al.^54^. Consequently, on the basis of the outcomes of Harman’s single-factor test, it was determined that there was no noteworthy common method bias among the variables.

### Validity analysis

Before validating the structural model, it was imperative to assess the validity of the questionnaire. This evaluation typically involves examining two key indicators, composite reliability (CR) and convergent validity, as indicated by the average variance extracted (AVE). CR serves as a robust measure of reliability, whereas AVE is a statistical metric used to assess the internal consistency and reliability of the constructs (latent variables). In confirmatory studies grounded in established theory, it is generally deemed acceptable for CR and AVE to surpass 0.7 and 0.5, respectively. Meeting these criteria suggests that the composite reliability and convergent validity of the items within the latent variables are satisfactory^55,56^. In this investigation, the values of these indicators were computed via SPSS 25.0 and Amos 24.0. The results revealed that the CR and AVE all exceeded the recommended thresholds (Table 2). These outcomes implied that the measurement items exhibited high reliability and strong internal consistency.

**Table 2:** Measurement model (convergent validity and reliability).

Constructs	Items	Significance estimation				Question reliability		CR	AVE
		UnStd.	S.E.	Z Value	*P*	Std.	SMC		
PU	PU4	1.000				0.801	0.642	0.873	0.633
	PU3	0.992	0.064	15.416	***	0.787	0.619		
	PU2	0.986	0.065	15.215	***	0.778	0.605		
	PU1	1.019	0.064	16.038	***	0.816	0.666		
PEU	PEU4	1.000				0.798	0.637	0.864	0.613
	PEU3	1.013	0.068	14.920	***	0.778	0.605		
	PEU2	1.019	0.067	15.136	***	0.789	0.623		
	PEU1	0.976	0.067	14.665	***	0.766	0.587		
TAC	TAC3	1.000				0.788	0.621	0.807	0.582
	TAC2	0.883	0.071	12.404	***	0.735	0.540		
	TAC1	0.938	0.074	12.709	***	0.765	0.585		
TEC	TEC3	1.000				0.750	0.563	0.819	0.601
	TEC2	0.985	0.076	12.934	***	0.786	0.618		
	TEC1	1.070	0.083	12.956	***	0.789	0.623		
SS	SS4	1.000				0.775	0.601	0.847	0.581
	SS3	0.962	0.073	13.220	***	0.727	0.529		
	SS2	0.963	0.068	14.066	***	0.773	0.598		
	SS1	1.027	0.073	14.042	***	0.772	0.596		
TTF	TTF1	1.000				0.771	0.594	0.807	0.583
	TTF2	0.964	0.077	12.544	***	0.746	0.557		
	TTF3	1.010	0.079	12.811	***	0.773	0.598		
CIS	CIS1	1.000				0.769	0.591	0.829	0.617
	CIS2	1.097	0.079	13.827	***	0.809	0.654		
	CIS3	1.027	0.076	13.511	***	0.778	0.605		

***p < 0.001.

Validity testing typically involves evaluating the convergent, content, and discriminant validity of observed variables. In this study, convergent validity was assessed through the AVE metric, ensuring that variables measuring the same construct converged. During the questionnaire design phase, content validity was established by initially deriving items from validated scales and refining them through the Delphi method and expert consultations to align with the study’s specific context. Discriminant validity, a focal point of our investigation, examines the unique correlations and distinctiveness among latent variables. This was evaluated by comparing the square root of the AVE with intervariable correlation coefficients, as per the criteria outlined by Fornell and Larcker^55^. For a variable to exhibit good discriminant validity, its correlation with other variables should be lower than the square root of its AVE. The bold values in Table 3 denote the square roots of the AVEs, which consistently surpass other values within their respective columns. Hence, the measurement model utilized in this research demonstrated satisfactory discriminant validity.

**Table 3:** Analysis of discriminant validity.

Constructs	AVE	Discriminant validity						
		CIS	FFT	SS	TEC	TAC	PEU	PU
CIS	0.617	**0.786**						
FFT	0.583	0.467	**0.763**					
SS	0.581	0.462	0.331	**0.762**				
TEC	0.601	0.445	0.363	0.343	**0.775**			
TAC	0.582	*0.448*	0.382	0.370	0.297	**0.763**		
PEU	0.613	0.492	0.382	0.453	0.337	0.366	**0.783**	
PU	0.633	0.506	0.389	0.371	0.376	0.355	0.356	**0.796**

The diagonals in bold are the square roots of AVE, and the lower triangle is the conformal Pearson correlation coefficient.

### Model goodness-of-fit tests

Due to the absence of standalone robust evaluation metrics like traditional methods such as ANOVA and regression, evaluating fit in structural equation modelling (SEM) often involves comparing sample covariance matrices with the theoretical model, leading to the creation of various fit indices^56^. The determination of fit indices in SEM is influenced by the specific research field and context, often drawing from established scholarly works by leading researchers. The criteria for fit indices can vary between confirmatory and exploratory studies, with exploratory research typically following less stringent standards. Different disciplines also have their own set of criteria. Table 4 displays the numerical values of the fit indices for the model in this study alongside the recommended values provided by respected scholars in the field. A comparative analysis indicated that the goodness-of-fit indices met the prescribed benchmarks, indicating that the model effectively captured the characteristics of the gathered data.

**Table 4:** Model fit indices.

Indices	Model index values	Standards	Conclusion	Reference
CMID	338.751	The smaller the better		
DF	239.000	The smaller the better		
CMID/DF	1.417	<3	Excellent fit	^57^
GFI	0.929	> 0.8 acceptable; >0.9 excellent fit	Excellent fit	^58^
AGFI	0.911	> 0.8 acceptable; >0.9 excellent fit	Excellent fit	^59^
CFI	0.974	> 0.9	Excellent fit	^58^
TLI(NNFI)	0.970	> 0.9	Excellent fit	^43^
RMSEA	0.035	< 0.08	Excellent fit	^43^
SRMR	0.066	< 0.08	Excellent fit	^60^

### Structural model validation

**Fig. 2:**
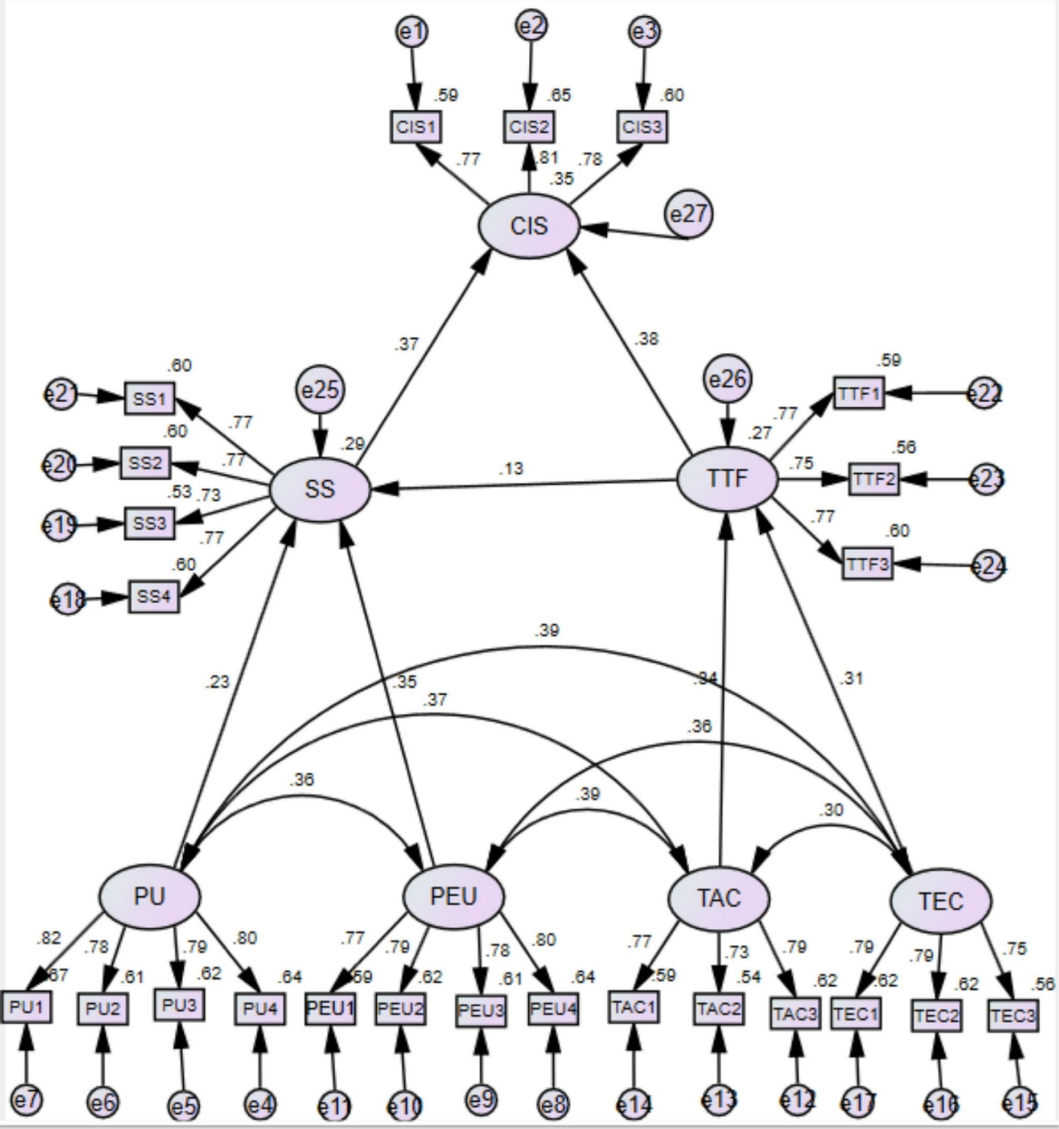
Diagram of the structural equation model. *PU* perceived usefulness, *PEU* perceived ease of use, *SS* students’ satisfaction, *TAC* task characteristics, *TEC* technology characteristics, *TTF* task–technology fit, *CIS* continuance intention towards simulation software.

The validation of the structural model involved estimating the path coefficients and the variance explained by each variable (R^2^) via Amos 24.0, as depicted in Fig. 2. The outcomes revealed that all seven hypotheses (H1 to H7) were corroborated, as indicated in Table 5.

**Table 5:** Results of model path analysis.

Assumption	UnStd.	S.E.	C.*R*.	*P*	Std. (β)	*R*	*R* ^2^
Task characteristics→Task–technology fit	0.330	0.066	5.022	***	0.337	0.520	0.270
Technology characteristics→Task–technology fit	0.315	0.068	4.662	***	0.309		
Perceived usefulness→Students’ satisfaction	0.207	0.057	3.618	***	0.226	0.535	0.286
Perceived ease of use→Students’ satisfaction	0.332	0.061	5.483	***	0.355		
Task–technology fit→Students’ satisfaction	0.120	0.057	2.093	0.036	0.127		
Students’ satisfaction→Continuance intention towards simulation software	0.381	0.065	5.894	***	0.368	0.595	0.354
Task–technology fit→Continuance intention towards simulation software	0.370	0.063	5.886	***	0.379		

As shown in Table 5, task characteristics (β = 0.337, *p* < 0.001) and technology characteristics (β = 0.309, *p* < 0.001) notably and positively impacted task‒technology fit. In addition, perceived usefulness (β = 0.226, *p* < 0.001) and perceived ease of use (β = 0.355, *p* < 0.001) significantly and positively influenced the satisfaction of mechatronic engineering students with simulation software. Moreover, task‒technology fit (β = 0.127, *p* < 0.05) positively influenced the students’ continuance intentions to use simulation software. Students’ satisfaction (β = 0.368, *p* < 0.001) and task‒technology fit (β = 0.379, *p* < 0.001) significantly influenced their continuance intentions to use simulation software. As a result, Hypotheses H1–H7 were effectively supported. In summary, the task characteristics and technology characteristics jointly accounted for 27% of the variance in task‒technology fit, and the explained variances in perceived usefulness, perceived ease of use, and task‒technology fit collectively explained 28.6% of the variance in students’ satisfaction. Students’ satisfaction and task‒technology fit accounted for 35.4% of the variance in the continuance intention to use simulation software.

## Discussion

### Factors influencing task‒technology fit in mechatronic engineering students’ use of simulation software

The findings revealed that task characteristics and technology characteristics impacted students’ use of simulation software in their mechatronic engineering studies. Task characteristics contribute 33.7% of the variance in task‒technology fit, and for technology characteristics, the number is 30.9%, which is slightly lower than that of task features, but the two are essentially equal. This finding indicates that the characteristics, nature, and goals of the learning and research tasks of students in the field of mechatronic engineering have a slightly greater impact on task‒technology fit than the features and capabilities of the simulation software itself. If task characteristics increased by one unit, task‒technology fit with simulation software would increase by 0.337 units, surpassing the impact of technology characteristics (0.028 units).

### Factors influencing students’ satisfaction with simulation software in mechatronic engineering education

The findings revealed that perceived usefulness, perceived ease of use, and task‒technology fit impacted students’ satisfaction with simulation software, contributing to 28.6% of the variance in students’ satisfaction. Further analysis of the path coefficients indicated that task‒technology fit had a path coefficient of 0.127, perceived usefulness had a path coefficient of 0.226, and perceived ease of use had a path coefficient of 0.355. This finding indicates that the perceived ease of using simulation software in mechatronic engineering education had the greatest influence on students’ satisfaction. If the ease of use of simulation software increased by one unit, students’ satisfaction would increase by 0.355 units, surpassing the impact of perceived ease of use (0.129 units). This suggested that in mechatronic engineering education, when students use simulation software for their learning and research, they are more concerned about the ease of use of simulation software rather than its usefulness.

Furthermore, task‒technology fit had a path coefficient of 0.127 in students’ satisfaction with simulation software in mechatronic engineering education, representing the lowest impact among all three factors. This finding indicated that although the alignment between the characteristics of the simulation software and the students’ learning tasks increased their satisfaction, it was not the most important factor in improving their satisfaction. This suggests that students majoring in mechatronic engineering differ from practitioners in the field of mechatronic engineering technology, who often have well-defined goals and distinctive task characteristics. One advantage of simulation software is that it provides users with the ability to verify the feasibility and effectiveness of design proposals. By simulating and analysing various engineering scenarios, electromechanical engineers can identify potential issues and enhance design proposals. Through simulation-based analysis via such software, electromechanical engineers can accurately predict performance indicators of electrical equipment, mechanical devices, or systems, such as power consumption, efficiency, and response time, enabling optimization during the design phase. However, students in the field of mechatronic engineering often do not have such clear task characteristics or such high design goals during their study process. During the process of using simulation software as their study tool, students are most concerned about the ease of use of the software, meaning that they believe that using simulation software will be effortless. Additionally, they pay attention to the usefulness of the simulation software, which refers to the extent to which a user believes that using a particular technology will enhance their performance or productivity. For students in the field of electromechanical engineering, perceived usefulness includes their belief that simulation software can enhance their learning ability and efficiency.

In summary, this analysis underscores that students’ satisfaction with simulation software in their mechatronic engineering study is the result of multiple influencing factors, including perceived usefulness, perceived ease of use, and task‒technology fit. Both perceived ease of use and perceived usefulness were identified as crucial factors that directly impacted the satisfaction of the students. Task‒technology fit has a significant effect on students’ satisfaction with and willingness to continue using simulation software. This alignment provides a crucial framework for better meeting students’ study needs. These insights not only offer valuable perspectives for the design and enhancement of simulation software but also present effective directions for the advancement of mechatronics engineering education.

### Factors influencing students’ continuance intentions in the use of simulation software in mechatronic engineering education

The analysis results from AMOS revealed that task‒technology fit and students’ satisfaction directly impacted the students’ continuance intentions to use simulation software, contributing to 35.4% of the variance in the students’ continuance intention. Further analysis of the path coefficients indicated that task‒technology fit (β = 0.379) had a slightly greater effect on students’ continuance intentions towards simulation software than students’ satisfaction (β = 0.368). This suggests that the effects of task‒technology fit and satisfaction on students’ willingness to persist in using the simulation software are approximately similar. The impact of task‒technology fit on students’ intention to continue using simulation software is slightly more significant than on their satisfaction. In other words, in the study of mechatronic engineering, if students believe that the simulation software effectively supports task completion or if they feel satisfied with the simulation software, it will increase their intention to continue using it. Among the two factors, the former has a slightly stronger influence than the latter.

In this study, task‒technology fit not only directly influenced students’ continuance intentions towards the simulation software in their mechatronic engineering learning but also indirectly affected the intentions through students’ satisfaction with the simulation software. By examining the path coefficient of task‒technology fit, the overall effect of the independent variable on the dependent variable can be calculated, providing a comprehensive understanding of its impact. In this study, the direct effect of task‒technology fit on the students’ continuance intentions was 0.379, whereas the indirect effect of satisfaction was 0.13 * 0.37 = 0.0481. The total effect was the sum of the direct and indirect effects, which was 0.379 + 0.0481 = 0.4271.

Our analysis of the total effect revealed that the overall impact of task‒technology fit on the students’ continuance intentions towards simulation software should not be overlooked. Although the match between task‒technology fit and students’ satisfaction with simulation software is not very significant, it significantly influences their willingness to continue using the simulation software. The reason is that for users at the stage of receiving education in mechatronic engineering, simulation software primarily serves as a learning tool. Therefore, students are particularly focused on whether they can quickly grasp how to use simulation software and whether it can enhance their learning abilities and academic performance. However, students predict that they will gradually need to handle more complex tasks, allowing them to anticipate the increased expertise and functional requirements of the tools they will use in the future. The distinctive characteristics of simulation software can provide users with powerful simulation analysis functions and simulation capabilities for various operating scenarios of complex systems, such as allowing users to create intricate models of electromechanical systems, including components such as sensors, actuators, circuits, and controllers; helping users simulate system failure scenarios to identify and resolve potential issues; and helping users evaluate the performance of electromechanical systems under various working conditions to guide improvements and optimization. These characteristics enable students to promptly address significant challenges within their assignments^13^.

In summary, these findings emphasize the importance of satisfaction as a driver of the continuance intention towards simulation software in students’ mechatronic engineering learning and research. Additionally, they underscore the dual role of task‒technology fit, which not only directly influences continuance intention but also operates indirectly through the mediation of students’ satisfaction. Therefore, simulation software should focus on ensuring the compatibility of technology characteristics with the demands of students’ study tasks, as these factors significantly contribute to overall student satisfaction and continuance intention towards simulation software in mechatronic engineering education.

## Theoretical contributions and practical implications

### Theoretical contributions

From an academic perspective, the present study aims to provide an empirical investigation and a nuanced understanding of students’ continuance intention to use simulation software in the context of mechatronic engineering education by integrating two seminal models: the technology acceptance model (TAM) and the task‒technology fit (TTF) model. Simulation software, with its novel attributes such as simulation capabilities for various operating scenarios of systems and simulation analysis functions, has gained widespread use as a learning and research tool for students in mechatronic engineering education. However, an in-depth exploration is needed to elucidate the underlying mechanisms and essential logic behind this novel tool of learning and research. Consequently, this study provides valuable theoretical contributions to the fields of mechatronic engineering education, the development and application of simulation laboratories, and technology acceptance in the following ways.

First, this study not only provides valuable insights by exploring the specific characteristics of simulation software but also elucidates the significance of perceived usefulness, perceived ease of use and task‒technology fit on students’ satisfaction with simulation software as a tool in mechatronic engineering education through an empirical investigation. To the best of our knowledge, the existing research has focused mainly on modelling methods based on various simulation software^11^ and the use of simulation software in mechanical engineering education^12^. There has been no prior research investigating students’ continuance intention towards simulation software in mechatronic engineering education, making the insights from this study a valuable extension at the theoretical level.

Second, this study validates the value and significance of integrating the TAM and TTF model in a new domain. This integrated model considers both technology acceptance factors (from the TAM) and the alignment between technology and tasks (from the TTF model). The theoretical contribution of existing research lies in the integration of these two concepts to provide a more comprehensive understanding of user behaviour in the context of education. For example, Yang discussed the continuance intention to use MOOCs^21^, and Wang examined the continuance intention of college students towards new e-learning spaces^44^. This study developed empirical research on the integration model of the TAM and TTF, providing new evidence for this type of research.

Third, this study emphasized the pivotal role of students’ satisfaction in their continuance intention to use simulation software in mechatronic engineering education. Previous studies have frequently identified perceived usefulness, perceived ease of use, and task‒technology fit as pivotal determinants that predominantly influence users’ adoption intentions through satisfaction^21,44^. As a result, learner satisfaction emerged as the central construct in the overall model. The investigation and examination of the mechanisms surrounding this fundamental variable have substantial implications for educators and researchers in the education domain.

### Practical implications

This research has significant practical value and offers guidance for designing simulation software, instructional strategies, user engagement and practitioners in the field of mechatronic engineering education. This study has the following practical implications for practitioners.

First, designers of simulation software should prioritize user-centric design, emphasising the perceived ease of use and perceived usefulness of the functionalities. It is crucial to integrate content with the various tasks that users may have and present them in a user-friendly manner. Additionally, simulation software should provide precise guidance in performing tasks for users, thereby increasing the ease of simulation software that students perceive. This approach is particularly essential in mechatronic engineering education, as students using simulation software for learning require easily comprehensible operational workflows and clear methods of operation. Improving the perceived ease of use and perceived usefulness in the design of simulation software can increase student satisfaction in mechatronic engineering education.

Second, developers of simulation software should consider enhancing the alignment of technical features with the study tasks of students in mechatronics engineering education. They should explore developing simulation software specifically tailored for the education sector, incorporating functionalities such as user-friendly interfaces, clear instructions, and intuitive design. These features, combined with analysis and simulation capabilities, would not only provide hands-on training in a controlled environment but also offer a platform for modelling and analysing complex systems and processes. This helps to provide a controlled setting to conduct experiments that allow students to predict outcomes, optimize designs, and make informed decisions. The above function should be further enhanced to improve students’ learning experience and increase the utilization of simulation software in the field of mechatronics engineering education.

Third, practitioners of mechatronics engineering education can incorporate the operation methods and usage skills of simulation software into the teaching content. This approach not only helps students better understand the working principle and performance optimization of mechanical and electrical systems but also allows them to acquire the basic operational skills of simulation software. It enables students to grasp simulation principles and methods, as well as how to apply simulation software for mechatronic system design, analysis, optimisation, and testing. By doing so, it enhances the perceived ease of use of simulation software among mechatronics engineering students, improves task‒technology fit, and ultimately increases students’ satisfaction and their continuance intention to use simulation software.

## Conclusions, limitations, and prospects

### Conclusions

This study empirically analyses satisfaction and the intention to continue using simulation software in the field of mechatronics engineering education, applying the TAM and TTF theory. A research model for the continued intention to use simulation software as a novel learning tool has been constructed and validated. The following conclusions were derived. First, the perceived ease of use variable from the TAM is a core factor influencing students’ satisfaction. The impact of perceived ease of use on students’ satisfaction was greater than that of perceived usefulness and the task‒technology fit variable from the TTF model. Second, task‒technology fit and student satisfaction both had significant positive effects on the continuance intention towards simulation software. Task‒technology fit not only directly impacts students’ continuance intention towards simulation software but also influences student satisfaction, indirectly affecting their continuance intention towards simulation software. Third, perceived ease of use and perceived usefulness have a more significant effect on students’ satisfaction with simulation software than does task‒technology fit. However, task‒technology fit has a more significant influence on students’ intention to continue using simulation software. This finding indicates that task‒technology fit plays a crucial role in students’ decisions on whether to continue using the simulation software; in other words, when deciding on the long-term use of simulation software, students are more concerned about whether the software can continuously meet their task needs. Fourth, upon analysing the model fit indices, the model integrating the TAM and TTF theory effectively predicted and elucidated students’ satisfaction with and intentions to continue using simulation software in mechatronics engineering education. This suggests that the task‒technology fit variable from the TTF theory complemented the TAM in this context.

These conclusions provide a support basis for further reform and development of simulation software, as well as the enhancement of mechatronics engineering education. It is essential to consider the perceived ease of use for students when designing and implementing simulation software, including its simulation capabilities and analytical functions in various operational scenarios. This ensures that the software aligns with the students’ study objectives and task characteristics, thereby enhancing their satisfaction with the simulation software. Furthermore, in mechanical and electrical engineering education, it is possible to incorporate the operation methods and usage skills of simulation software into the teaching content.

### Limitations and prospects

The current study has several limitations. First, the research did not account for the influence of individual capacities, social factors, or other variables on students’ satisfaction with and intentions to continue using simulation software in mechatronics engineering education. Second, the participant selection was restricted to students from universities in Shaanxi Province, limiting the generalizability of the findings given that the target audience for simulation software comprises students majoring in mechatronics engineering from diverse countries and regions. Future research should encompass a more diverse participant pool to improve the applicability of the study’s outcomes.

## Electronic supplementary material

Below is the link to the electronic supplementary material.

Supplementary Material 1

## Data Availability

Data is provided within the manuscript or supplementary information files.All the data generated or analysed in this study are included in the supplementary information file.

## References

[1] Abbas, A., Din, Z. U. & Farooqui, R. Integration of BIM in Construction Management Education: an overview of Pakistani Engineering universities. *Procedia Eng.* **145**, 151–157 (2016).

[2] Campos, N., Nogal, M., Caliz, C. & Juan, A. A. Simulation-based education involving online and on-campus models in different European universities. *Int. J. Educational Technol. High. Educ.* **17**, 8 (2020).

[3] Tanir, O. Simulation-based Software Engineering. in Guide to Simulation-Based Disciplines: Advancing Our Computational Future (eds Mittal, S., Durak, U. & Ören, T.) 151–166 (Springer International Publishing, Cham, doi:10.1007/978-3-319-61264-5_7. (2017).

[4] Mandal, S. Some important Simulation Software Tools for a student of Electronics Engineering. *Global J. Advancement Eng. Sci.* **3**, 1–8 (2017).

[5] *Advances in Manufacturing II: Volume 3 - Quality Engineering and Management*. (Springer International Publishing, Cham, (2019). 10.1007/978-3-030-17269-5

[6] Synthesis Lectures on Mechanical Engineering. *Springer* https://www.springer.com/series/16910

[7] Xue, D. & Chen, Y. *System Simulation Techniques with MATLAB and Simulink* (Wiley, 2014).

[8] Abbiati, G., Broccardo, M., Abdallah, I., Marelli, S. & Paolacci, F. Seismic fragility analysis based on artificial ground motions and surrogate modeling of validated structural simulators. *Earthq. Eng. Struct. Dynamics*. **50**, 2314–2333 (2021).

[9] Pressmair, N. et al. Bridging the gap between mathematical optimization and structural engineering: design, experiments and numerical simulation of optimized concrete girders. *Struct. Concrete*. **24**, 5314–5330 (2023).

[10] Muramatsu, D. & Sasaki, Y. 2.4 GHz/5.6 GHz Dual-Use Wearable Patch Antenna Integrated with electrodes and parasitic element for Wireless Body Area Network. *IEEJ Trans. Electr. Electron. Eng.* **19**, 154–156 (2024).

[11] Rahman, M. A. A. & Mizukawa, M. Modeling and design of mechatronics system with SysML, Simscape and Simulink. 10.1109/AIM.2013.6584353

[12] Kenjo, T., Kikuchi, T. & Kubo, M. Developing educational software for mechatronics simulation. *IEEE Trans. Educ.* **44**, 29 (2001).

[13] Negahban, A. Simulation in engineering education: the transition from physical experimentation to digital immersive simulated environments. *SIMULATION* **00375497241229757**10.1177/00375497241229757 (2024).

[14] Davis, F. D. & Perceived Usefulness Perceived ease of Use, and user Acceptance of Information Technology. *MIS Q.* **13**, 319–340 (1989).

[15] Davis, F. D., Bagozzi, R. P. & Warshaw, P. R. User Acceptance of Computer Technology: a comparison of two theoretical models. *Manage. Sci.* **35**, 982–1003 (1989).

[16] Bazelais, P., Doleck, T. & Lemay, D. J. Investigating the predictive power of TAM: a case study of CEGEP students’ intentions to use online learning technologies. *Educ. Inf. Technol.* **23**, 93–111 (2018).

[17] Jones, S. M., Bouffard, S. M. & Weissbourd, R. Educators’ Social and emotional skills vital to learning. *Phi Delta Kappan*. **94**, 62–65 (2013).

[18] Fayad, R. & Paper, D. The Technology Acceptance Model E-Commerce extension: a conceptual Framework. *Procedia Econ. Finance*. **26**, 1000–1006 (2015).

[19] Holden, R. J. & Karsh, B. T. The Technology Acceptance Model: its past and its future in health care. *J. Biomed. Inform.* **43**, 159–172 (2010). 19615467 10.1016/j.jbi.2009.07.002 PMC2814963

[20] Garcia, M. B. Factors affecting adoption intention of Productivity Software Applications among teachers: a structural equation modeling investigation. *Int. J. Human–Computer Interact.* **40**, 2546–2559 (2024).

[21] Yang, M., Shao, Z., Liu, Q. & Liu, C. Understanding the quality factors that influence the continuance intention of students toward participation in MOOCs. *Educ. Tech. Res. Dev.* **65**, 1195–1214 (2017).

[22] Marques, B. P., Villate, J. E. & Carvalho, C. V. Applying the UTAUT model in Engineering Higher Education: Teacher’s technology adoption. in *6th Iberian Conference on Information Systems and Technologies (CISTI* 1–6 (2011). (2011).

[23] Goodhue, D. L. & Thompson, R. L. Task-Technology Fit and Individual Performance. *MIS Q.* **19**, 213 (1995).

[24] Parkes, A. The effect of task–individual–technology fit on user attitude and performance: an experimental investigation. *Decis. Support Syst.* **54**, 997–1009 (2013).

[25] Zhou, T., Lu, Y. & Wang, B. Integrating TTF and UTAUT to explain mobile banking user adoption. *Comput. Hum. Behav.* (2010).

[26] Aljukhadar, M., Senecal, S. & Nantel, J. Is more always better? Investigating the task-technology fit theory in an online user context. *Inf. Manag.* **51**, 391–397 (2014).

[27] Rai, R. S. & Selnes, F. Conceptualizing task-technology fit and the effect on adoption – a case study of a digital textbook service. *Inf. Manag.* **56**, 103161 (2019).

[28] Raven, A., Leeds, E. & Park, C. Digital Video Presentation and Student Performance: a Task Technology Fit Perspective. *Int. J. Inform. Communication Technol. Educ. (IJICTE)*. **6**, 17–29 (2010).

[29] Ursavaş, Ö. F. Unified theory of Acceptance and Use of Technology Model (UTAUT). in Conducting Technology Acceptance Research in Education: Theory, Models, Implementation, and Analysis (ed Ursavaş, Ö. F.) 111–133 (Springer International Publishing, Cham, doi:10.1007/978-3-031-10846-4_6. (2022).

[30] Determinants of Technology Acceptance. Two Model-Based Meta-Analytic Reviews. https://journals.sagepub.com/doi/epub/10.1177/1077699020952400 doi:10.1177/1077699020952400.

[31] Alsabawy, A. Y., Cater-Steel, A. & Soar, J. Determinants of perceived usefulness of e-learning systems. *Comput. Hum. Behav.* **64**, 843–858 (2016).

[32] He, Y., Chen, Q. & Kitkuakul, S. Regulatory focus and technology acceptance: perceived ease of use and usefulness as efficacy. *Cogent Bus. Manage.* **5**, 1459006 (2018).

[33] Al-Emran, M., Mezhuyev, V. & Kamaludin, A. Towards a conceptual model for examining the impact of knowledge management factors on mobile learning acceptance. *Technol. Soc.* **61**, 101247 (2020).

[34] Taherdoost, H. A review of technology acceptance and adoption models and theories. *Procedia Manuf.* **22**, 960–967 (2018).

[35] Thong, J. Y. L. & Yap, C. S. Information systems effectiveness: a user satisfaction approach. *Inf. Process. Manag.* **32**, 601–610 (1996).

[36] Pozón-López, I., Higueras-Castillo, E., Muñoz-Leiva, F. & Liébana-Cabanillas, F. J. Perceived user satisfaction and intention to use massive open online courses (MOOCs). *J. Comput. High. Educ.* **33**, 85–120 (2021).

[37] Oliver, R. L. A cognitive model of the antecedents and consequences of satisfaction decisions. *J. Mark. Res.* **17**, 460–469 (1980).

[38] Pal, D. & Patra, S. University Students’ perception of video-based learning in Times of COVID-19: a TAM/TTF perspective. *Int. J. Human–Computer Interact.* **37**, 903–921 (2021).

[39] Gan, C., Li, H. & Liu, Y. Understanding mobile learning adoption in higher education: an empirical investigation in the context of the mobile library. *Electron. Libr.* **35**, 846–860 (2017).

[40] Oliveira, T., Faria, M., Thomas, M. A. & Popovič, A. Extending the understanding of mobile banking adoption: when UTAUT meets TTF and ITM. *Int. J. Inf. Manag.* **34**, 689–703 (2014).

[41] Hsiao, J. L. & Chen, R. F. An investigation on task-technology fit of mobile nursing information systems for nursing performance. *Comput. Inf. Nurs.* **30**, 265–273 (2012). 10.1097/NCN.0b013e31823eb82c 22156768

[42] Larsen, T. J., Sørebø, A. M. & Sørebø, Ø. The role of task-technology fit as users’ motivation to continue information system use. *Comput. Hum. Behav.* **25**, 778–784 (2009).

[43] Hair, J. F. Jr., Babin, B. J. & Krey, N. Covariance-based structural equation modeling in the Journal of Advertising: review and recommendations. *J. Advertising*. **46**, 163–177 (2017).

[44] Wang, C., Dai, J., Zhu, K., Yu, T. & Gu, X. Understanding the Continuance Intention of College Students toward New E-Learning spaces based on an Integrated Model of the TAM and TTF. *Int. J. Human–Computer Interact.* **0**, 1–14 (2023).

[45] Batool, S. H., Rehman, A. & Sulehri, I. The current situation of information literacy education and curriculum design in Pakistan: a discovery using Delphi method. *Libr. Hi Tech.* **40**, 1705–1720 (2021).

[46] Hair, J. F., Black, W. C., Babin, B. J. & Anderson, R. E. *Multivariate Data Analysis*. (Cengage, Andover, Hampshire, (2019).

[47] Sukendro, S. et al. Using an extended Technology Acceptance Model to understand students’ use of e-learning during Covid-19: Indonesian sport science education context. *Heliyon* **6**, e05410 (2020). 33195843 10.1016/j.heliyon.2020.e05410 PMC7644906

[48] Salloum, S. A., Mohammad Alhamad, Q., Al-Emran, A., Abdel Monem, M., Shaalan, K. & A. & Exploring students’ Acceptance of E-Learning through the development of a Comprehensive Technology Acceptance Model. *IEEE Access.* **7**, 128445–128462 (2019).

[49] Cheng, Y. M. How does task-technology fit influence cloud-based e-learning continuance and impact? *Educ. + Train.* **61**, 480–499 (2019).

[50] 登录页面_问卷星. https://www.wjx.cn/login.aspx

[51] Barrett, P. Structural equation modelling: adjudging model fit. *Pers. Indiv. Differ.* **42**, 815–824 (2007).

[52] Cham, T. H., Cheng, B. L., Low, M. P. & Cheok, J. B. C. Brand image as the competitive edge for hospitals in medical tourism. *Eur. Bus. Rev.* **33**, (2020).

[53] Shiau, W. L., Sarstedt, M. & Hair, J. F. Internet research using partial least squares structural equation modeling (PLS-SEM). *Internet Res.* **29**, 398–406 (2019).

[54] Hair Jr, F., Sarstedt, J., Hopkins, M., Kuppelwieser, G. & L. & V. partial least squares structural equation modeling (PLS-SEM): an emerging tool in business research. *Eur. Bus. Rev.* **26**, 106–121 (2014).

[55] Fornell, C. & Larcker, D. F. Evaluating Structural equation models with unobservable variables and measurement error. *J. Mark. Res.* **18**, 39–50 (1981).

[56] Hair, J. F., Sarstedt, M., Ringle, C. M. & Mena, J. A. An assessment of the use of partial least squares structural equation modeling in marketing research. *J. Acad. Mark. Sci.* **40**, 414–433 (2012).

[57] Hayduk, L. A. *Structural Equation Modeling with LISREL: Essentials and Advances* (Johns Hopkins Univ., 1995).

[58] Bagozzi, R. P. & Yi, Y. On the evaluation of structural equation models. *JAMS* **16**, 74–94 (1988).

[59] Scott, J. E. The measurement of information systems effectiveness: evaluating a measuring instrument. *SIGMIS Database*. **26**, 43–61 (1995).

[60] Hu, L. & Bentler, P. M. Fit indices in covariance structure modeling: sensitivity to underparameterized model misspecification. *Psychol. Methods*. **3**, 424–453 (1998).

